# Vaccine–derived Poliovirus, Thailand, 2003

**DOI:** 10.3201/eid1105.040528

**Published:** 2005-05

**Authors:** Piyanit Tharmaphornpilas, for the Outbreak Response Team, Thailand

**Affiliations:** * Ministry of Public Health, Nonthaburi, Thailand

**Keywords:** Poliovirus, Polio Eradication, iVDPV/immunodeficiencies vaccinederived poliovirus, outbreak response immunization

**To the Editor:** The Polio Eradication Campaign was started in Thailand in 1990, and the last polio case was reported in April 1997. Although no new cases have been reported, the Polio Eradication Campaign continues with 4 prevention strategies: high coverage with 3 doses of oral polio in children <1 year of age, acute flaccid paralysis surveillance, acute flaccid paralysis case investigation and response, and National Immunization Day. Also, the Ministry of Public Health is prepared for a national emergency response to polio importation and circulated vaccine–derived poliovirus (1).

In April 2003, a case of acute flaccid paralysis was reported from Phakhao district, Loei province. The patient was an 18-month-old boy with normal physical development and nutritional status. He had a history of mild asthma and had received bronchodilator drugs occasionally during upper respiratory tract infections in the past. The patient had been fully vaccinated. He had received a total of 5 doses of oral polio vaccine: a dose at 2, 4, and 6 months of age, and 2 doses on National Immunization Day in December 2002 and January 2003.

On March 27, 2003, while visiting his grandmother in Phoowiang district, Khonkaen province (80 km from his residence), the patient became ill. Pneumonia was diagnosed; injected medications were administered into his left hip once a day for 3 days. The patient fully recovered.

On April 1, 2003, cellulitis of finger developed in the patient. The affected finger was incised and drained, and oral antimicrobial drugs were administered. The inflammation extended to his elbow but later subsided.

On April 7, fever, cough, and dyspnea developed in the patient. Two days later, the patient's left leg became weak. He was admitted to Phakhao Hospital with a diagnosis of pneumonia and with weakness in his left leg. He was later referred to Loei Provincial Hospital and acute flaccid paralysis was diagnosed on April 11. The muscle weakness progressed until he could not sit.

On April 14, the patient was referred to Khonkaen Regional Hospital with weakness in both legs and arms (grade 0–1). Chest radiograph showed perihilar pneumonia. Cloxacillin, gentamicin, and immunoglobulin (Ig) (6 g/day × 4 days, patient weight 12 kg) were administered intravenously to the patient. He was discharged on April 30 with a diagnosis of Guillain-Barré syndrome and bacterial pneumonia. The muscle tone in his right leg and both arms was grade 3; however, he could not move his left leg.

Stool samples were collected on April 11 and 14 and tested for polio at the Department of Medical Science (reference laboratory for polio in Southeast Asia). Poliovirus type 2 was isolated in the samples; however, the results were inconclusive for strain differentiation. The isolates were sent to the Centers for Disease Control and Prevention (CDC), USA, for genetic sequencing, and the result showed poliovirus type 2 with 1.6% difference from Sabin strain poliovirus. Without evidence of recombination with other nonpolio enterovirus, the pattern of genomic change was similar to the change that occurs in immunodeficient persons. Immune system testing of the patient on August 13 showed IgG = 205.9 mg/dL (normal 800–1,700), IgA <5.5 mg/dL (normal 100–490), IgM <16.8 mg/dL (normal 50–320), and IgE <18.0 mg/dL (normal 0–100). Antibodies to poliovirus type 1, 2, and 3 were 1:16, 1:32, and 1:8, respectively. Testing of the follow-up stool samples showed P1/Sabin on August 10. Test results were negative on October 13, and results showed nonpolio enterovirus on November 10 and December 14.

Before the large-scale outbreak response immunization was conducted, 339 serum samples were collected from children <5 years of age who lived in the same district as the patient or in the same subdistrict as his grandmother. Among 153 children who brought their vaccination records, the median dose of oral polio vaccine was 7 (range 2–15). All had antibody >1:8 to poliovirus types 1, 2, and 3. Approximately 2,000 stool samples were collected from children <5 year of age who lived in the same district as the case-patient or his grandmother. However, after the immunodeficiencies vaccine–derived poliovirus was identified, isolation of the virus was attempted only from stool samples from children who lived in the same subdistrict as the patient. From 223 stool samples, 4 Sabin strain poliovirus and 32 nonpolio enteroviruses were isolated. In addition, 2 of 18 stool samples collected in July from close contacts of the case-patient were positive for Sabin strain poliovirus and negative for vaccine–derived poliovirus.

The Loei Provincial Health Office initially did a small-scale response immunization in 3 adjacent villages (128 of 129 children) on the day that the case of acute flaccid paralysis was reported. Coverage of third dose of oral polio vaccine in these villages was 100%. No response immunization was conducted at the village in Khonkaen. On August 8, genetic sequencing results showed vaccine–derived poliovirus; the decision was made to launch an outbreak response immunization for 175,000 children <5 years of age living in Loei, Khonkaen, and Nongbualampoo provinces (visited by the patient from March to August 2003). Two-round campaigns were conducted in August and September. The estimated vaccine coverage was >95%.

Considering the rate of 1% genomic diversity per year and the immunodeficient status of the patient (2), he should have harbored the vaccine strain virus since he received the first dose of routine oral polio vaccine immunization at 2 months of age, and the virus was replicated in his gut. However, why the virus disappeared in subsequent stool specimens is unknown. Circulating vaccine–derived poliovirus is unlikely in this event, as we found no evidence of recombination with other nonpolio enterovirus, high oral polio vaccine coverage in the community, and no vaccine–derived poliovirus in other children.

Although immunoglobulin levels in this case were low but still detectable, whether the patient's illness was agammaglobulinemia or hypogammaglobulinemia is uncertain. The detected immunoglobulin levels, as well as the antibody level to poliovirus, may be due to intravenous immunoglobulin (IVIG) the patient received while hospitalized 4 months before testing. Since August 2003, the patient has been on IVIG replacement therapy after prolonged and repeated respiratory tract infections.

In retrospect, problems surrounded this event. First, because of several attempts to confirm the result, identification of strain differentiation was delayed. Second, genetic sequencing was delayed because of a communication gap associated with new bioterrorism regulations in the United States during specimen transfer. Third, knowledge of a possible immune deficiency in the previously healthy child was lacking, testing for the patient's immune status was delayed.
